# Invasive Cancer Incidence — United States, 2009

**Published:** 2013-02-22

**Authors:** Simple Singh, S. Jane Henley, Reda Wilson, Jessica King, Christie Eheman

**Affiliations:** Div of Cancer Prevention and Control, National Center for Chronic Disease Prevention and Health Promotion, CDC

Cancer is a leading cause of illness and death in the United States, and many cancers are preventable (1). Surveillance of cancer incidence can help public health officials target areas for cancer control efforts (2) and track progress toward the national cancer objectives set forth in *Healthy People 2020* (3). This report summarizes the most recent invasive cancer incidence rates by sex, age, race, ethnicity, primary site, and state of residence using data from U.S. Cancer Statistics (USCS) for 2009. USCS includes incidence data from CDC’s National Program of Cancer Registries (NPCR) and the National Cancer Institute’s (NCI’s) Surveillance, Epidemiology, and End Results (SEER) program and mortality data from the National Vital Statistics System (4). In 2009, a total of 1,476,504 invasive cancers were diagnosed in the United States, an annual incidence rate of 459 cases per 100,000 persons. Cancer incidence rates were higher among men (524) than women (414), highest among blacks (473) and lowest among American Indian/Alaska Natives (273), and ranged by state from 387 to 509. Populations defined by state of residence, race, or ethnicity with high rates of cancer might benefit most from targeted cancer prevention and control efforts.

Data on new cases of invasive cancer diagnosed during 2009 were obtained from population-based cancer registries affiliated with the NPCR and SEER programs. Invasive cancers are all cancers except in situ cancers (except in the urinary bladder) or basal and squamous cell skin cancers. In each state and the District of Columbia (DC), data about new diagnoses of cancer are collected from patient records at hospitals, physicians’ offices, therapeutic radiation facilities, freestanding surgical centers, and pathology laboratories and reported to NPCR or SEER central cancer registries. The central cancer registries collate these data and use state vital records, the Social Security Index, and the National Death Index to collect information about any cancer deaths that were not reported as cases. These data are submitted to CDC or NCI and combined into one dataset by CDC (4). Data from all cancer registries met the six USCS publication criteria for 2009.* For this report, however, data from Wisconsin for 2009 were suppressed at that state’s request. A central cancer registry may request time for making corrections and may suppress their data for various reasons. With the exclusion of data from Wisconsin, data in this report cover 98% of the U.S. population.

Cases were classified by site using the *International Classification of Diseases for Oncology, Third Edition* (ICD-O-3). Breast cancers also were characterized by stage at diagnosis using SEER Summary Stage 2000†; late-stage cancers include those diagnosed at a regional or distant stage.

Race and ethnicity information was abstracted from medical records. Race was categorized as white, black, American Indian/Alaska Native, or Asian/Pacific Islander. Ethnicity was categorized as Hispanic or non-Hispanic.

Postcensal population denominators for incidence rates were race-specific, ethnicity-specific, and sex-specific county population estimates from the 2000 U.S. Census, as modified by SEER and aggregated to the state and national level.§ Annual incidence rates per 100,000 population were age-adjusted by the direct method to the 2000 U.S. standard population.

In 2009, a total of 1,476,504 invasive cancers were diagnosed and reported to central cancer registries in the United States (excluding Wisconsin), including 757,545 among males and 718,959 among females (Table). The age-adjusted annual incidence for all cancers was 459 per 100,000 population (524 per 100,000 in males and 414 per 100,000 in females). Among persons aged ≤19 years, 14,023 cancer cases were diagnosed in 2009 (Table). By age group, rates per 100,000 population in 2009 were 16.9 among persons aged ≤19 years, 155.5 among those aged 20–49 years, 843.2 among those aged 50–64 years, 1,903.0 among those aged 65–74 years, and 2,223.0 among those aged ≥75 years (Table).

What is already known on this topic?

Cancer is a leading cause of illness and death in the United States, and many cancers are preventable.

What is added by this report?

National cancer surveillance data indicate that 1,476,504 new cases of invasive cancer were diagnosed in the United States in 2009, an annual incidence rate of 524 cases per 100,000 among men and 414 among women. Rates were highest (473 per 100,000 population) among blacks and lowest among American Indian/Alaska Natives (273), largely reflecting differences in rates of cancers of the prostate and female breast. By state, all-sites cancer incidence rates ranged from 387 to 509 per 100,000 population. The *Healthy People 2020* objective for reduced incidence of colorectal cancer was met among women and in some states.

What are the implications for public health practice?

High rates of cancer by race, ethnicity, and state of residence indicate populations that might benefit most from targeted cancer prevention and control efforts. National cancer surveillance data help public health officials track progress toward the national cancer objectives set forth in *Healthy People 2020*.

By cancer site, rates were highest for cancers of the prostate (137.7 per 100,000 men), female breast (123.1 per 100,000 women), lung and bronchus (64.3 overall, 78.2 among men and 54.1 among women), and colon and rectum (42.5 overall, 49.2 among men and 37.1 among women) (Table). These four sites accounted for half of cancers diagnosed in 2009, including 206,640 prostate cancers, 211,731 female breast cancers, 205,974 lung and bronchus cancers (110,190 among men and 95,784 among women), and 136,717 colon and rectum cancers (70,223 among men and 66,494 among women).

The top 10 cancer sites differed by sex and racial and ethnic group (Figure 1). Among men in 2009, prostate cancer was the most common cancer in all racial and ethnic groups; lung and colorectal cancers were the second and third most common cancers in all racial and ethnic groups, except among Hispanic men, among whom the order was switched. Among women in 2009, breast cancer was the most common cancer among all racial and ethnic groups, followed by lung, colorectal, and uterine cancers in all racial and ethnic groups, except among Hispanic women, among whom colorectal cancer was more common than lung cancer, and Asian/Pacific Islander women, among whom the most common cancers were colorectal, lung, and thyroid (Figure 1). Beyond these cancers, cancer ranking varied by race and ethnicity. Incidence of late-stage breast cancer was highest among black women (Figure 1).

By state in 2009, all-sites cancer incidence rates ranged from 387.1 per 100,000 population to 509.1 (Figure 2). State site-specific cancer incidence rates ranged from 95.2 to 178.4 for prostate cancer, 104.7 to 139.2 for female breast cancer, 28.1 to 96.9 for lung cancer, and 30.8 to 52.8 for colorectal cancer (Figure 2).

## Editorial Note

Twenty years ago, Congress established NPCR by enacting the Cancer Registries Amendment Act (Public Law 102–515) to ensure that state cancer registries are population-based and meet minimum standards of completeness, timeliness, and quality (5). This act authorized CDC to provide funds to states and territories to improve existing cancer registries; plan and implement registries where they do not exist; develop model legislation and regulations for states to enhance the viability of registry operations; set standards for data completeness, timeliness, and quality; provide training for registry personnel; and help establish a computerized reporting and data processing system (5). Before NPCR was established, 10 states had no cancer registry, and most states with registries lacked the resources and legislative authority needed to gather complete data (6). Today, NPCR supports central cancer registries in 45 states, DC, Puerto Rico, and the U.S. Affiliated Pacific Islands.

*Healthy People 2020* objectives call for increasing the number of central, population-based registries that capture case information on at least 95% of the expected number of reportable cancers (3). In 2011, 42 registries met this objective.

Data from population-based central cancer registries are essential for monitoring trends over time and identifying variations in rates by population factors such as age, race, ethnicity, or geographic region. This information can be useful in several ways. First, this information can guide the planning and evaluation of cancer prevention and control programs. The South Carolina Central Cancer Registry, for example, collaborated with comprehensive cancer control staff members and a regional health educator to present county-level information about cancer incidence, risk factors, and screening to the community.¶ Second, this information can assist long-term planning for adequate cancer diagnostic and treatment services. In Massachusetts, for example, cancer registry data will be used to evaluate the effect of universal health insurance on cancer treatment. Third, this information can help public health officials set priorities for allocating health resources and track progress toward the national goals and objectives regarding cancer set forth in *Healthy People 2020*. To address disparities in breast and cervical cancer in Mississippi, for example, cancer registry data are used to determine areas where interventions are needed most.

*Healthy People 2020* objectives call for reducing colorectal cancer incidence to 38.6 per 100,000 population, reducing late-stage breast cancer incidence to 41.0 per 100,000 women, and reducing cervical cancer incidence to 7.1 per 100,000 women (3). This report shows that the objective for reduced colorectal cancer incidence has been achieved among women and in some states. To reduce cancer incidence and achieve *Healthy People 2020* targets, evidence-based interventions can be implemented at both the individual level and the population level to reduce cancer risk factors, promote healthy living, and encourage colorectal, breast, and cervical cancer screening.

One of CDC’s goals is to provide high quality NPCR data via several data release products each year to public health officials and others for use in public health planning. These products include USCS, CDC WONDER, State Cancer Profiles, and National Center for Health Statistics (NCHS) Research Data Centers.** USCS is a joint publication from CDC and NCI in collaboration with the North American Association of Central Cancer Registries and contains the official federal government cancer incidence and mortality statistics for the U.S. population and for individual states. CDC WONDER is an online query system that produces tables, charts, and maps containing age-adjusted and crude rates by demographic variables. State Cancer Profiles brings together data collected from public health surveillance systems, including county-level data from NPCR. Restricted data from NPCR (and other datasets) are available through the Research Data Center hosted by CDC’s NCHS.

The findings in this report are subject to at least three limitations. First, postcensal populations for 2009 were estimated from the 2000 U.S. Census by the U.S. Census Bureau; errors in these estimates might increase as time passes after the census, leading to underestimates or overestimates of incidence rates (7). Second, analyses based on race and ethnicity might be biased if race and ethnicity were misclassified; efforts were made to ensure that this information was as accurate as possible.†† Finally, delays in cancer reporting might result in an underestimate of certain cancers; reporting delays are more common for cancers such as melanoma that are diagnosed and treated in nonhospital settings such as physicians’ offices (8).

Population-based central cancer registries provide cancer incidence surveillance critical to monitoring the cancer burden in the United States. These data can identify populations with high cancer rates that might benefit most from targeted cancer prevention and control efforts. National cancer surveillance data help public health officials track progress toward the national cancer objectives set forth in *Healthy People 2020*.

## Figures and Tables

**FIGURE 1:**
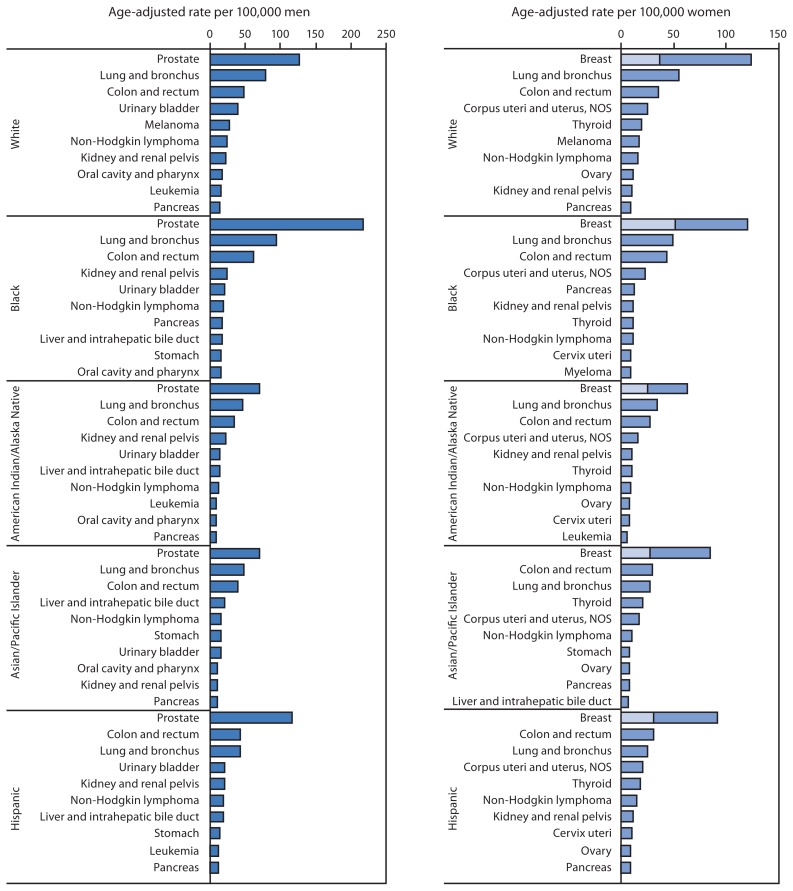
Invasive cancer incidence rates* for 10 primary sites^†^ with the highest rates within racial and ethnic groups,^§^ by sex — National Program of Cancer Registries (NPCR) and Surveillance, Epidemiology, and End Results (SEER) program,^¶^ United States, 2009 **Abbreviation:** NOS = not otherwise specified. * Rates are age-adjusted to the 2000 U.S. standard population. ^†^ Incidence of late-stage breast cancer is shown as a subset in bar for overall breast cancer incidence. ^§^ Race categories are not mutually exclusive from Hispanic ethnicity. ^¶^ Compiled from cancer registries that meet the data-quality criteria for all invasive cancer sites combined, covering approximately 98% of the U.S. population. Excludes basal and squamous cell carcinomas of the skin except when these occur on the skin of the genital organs, and in situ cancers except urinary bladder.

**FIGURE 2:**
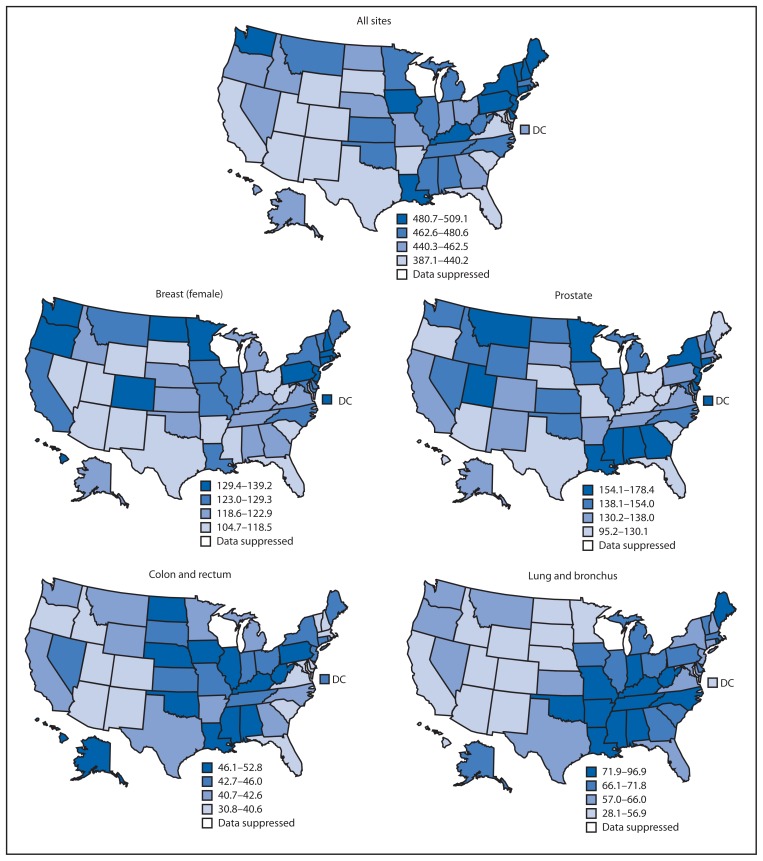
Invasive cancer incidence per 100,000 population, by primary cancer site — National Program of Cancer Registries (NPCR) and Surveillance, Epidemiology, and End Results (SEER) program, United States, 2009* * Age-adjusted to the 2000 U.S. standard population.

**TABLE:** Number and incidence* of invasive cancers,† by sex, primary sites, racial and ethnic group,§ and age group — National Program of Cancer Registries (NPCR) and Surveillance, Epidemiology, and End Results (SEER) program,¶ United States, 2009

	Overall			Men			Women		
			
Characteristic	Rate	No.	(%)	Rate	No.	(%)	Rate	No.	(%)
**All cancers**	**459.0**	**1,476,504**		**523.5**	**757,545**		**414.3**	**718,959**	
Prostate	NA	206,640	(14)	137.7	206,640	(27)	NA	NA	
Female breast	NA	211,731	(14)	NA	NA		123.1	211,731	(29)
Lung and bronchus	64.3	205,974	(14)	78.2	110,190	(15)	54.1	95,784	(13)
Colon and rectum	42.5	136,717	(9)	49.2	70,223	(9)	37.1	66,494	(9)
**Racial and ethnic group**									
White	456.5	1,244,503	(84)	513.0	636,138	(84)	418.2	608,365	(85)
Black	472.9	156,869	(11)	593.7	81,670	(11)	393.4	75,199	(10)
American Indian/Alaska Native	272.9	6,997	(<1)	294.8	3,427	(<1)	258.3	3,570	(<1)
Asian/Pacific Islander	291.8	39,213	(3)	309.6	17,820	(2)	283.5	21,393	(3)
Hispanic	353.0	102,278	(7)	395.2	50,074	(7)	327.9	52,204	(7)
**Age group (yrs)**									
≤19	16.9	14,023	(1)	17.7	7,481	(1)	16.2	6,542	(1)
20–49	155.5	192,055	(13)	114.8	71,622	(9)	196.3	120,433	(17)
50–64	843.2	477,087	(32)	924.4	254,091	(34)	768.2	222,996	(31)
65–74	1902.5	385,233	(26)	2368.2	220,684	(29)	1506.3	164,549	(23)
≥75	2223.3	408,106	(28)	2872.4	203,667	(27)	1810.9	204,439	(28)

**Abbreviation:** NA = not available.

* Age-adjusted to the 2000 U.S. standard population.

† Excludes basal and squamous cell carcinomas of the skin, except when these occur on the skin of the genital organs, and in situ cancers, except urinary bladder.

§ Race categories are not mutually exclusive from Hispanic ethnicity. Rates are not presented for cases with unknown or other race.

¶ Compiled from cancer registries that meet the data-quality criteria for all invasive cancer sites combined (covering approximately 98% of the U.S. population).

## Footnotes

* Cancer registries demonstrated that cancer incidence data were of high quality by meeting the six USCS publication criteria: 1) case ascertainment is ≥90% complete, 2) ≤5% of cases are ascertained solely on the basis of a death certificate, 3) ≤3% of cases are missing information on sex, 4) ≤3% of cases are missing information on age, 5) ≤5% of cases are missing information on race, and 6) ≥97% of the registry’s records passed a set of single-field and inter-field computerized edits that test the validity and logic of data components. Additional information available at http://www.cdc.gov/uscs.

† Additional information is available at http://seer.cancer.gov/tools/ssm.

§ Population estimates for 2009 incorporate bridged single-race estimates that are derived from the original multiple race categories in the U.S. 2000 Census. Additional information is available at http://seer.cancer.gov/popdata/index.html and http://www.census.gov/popest/topics/methodology.

¶ Additional information available at http://www.cdc.gov/cancer/npcr/success/index.htm.

** Additional information available at http://www.cdc.gov/cancer/npcr/datarelease.htm, http://wonder.cdc.gov/, http://www.statecancerprofiles.cancer.gov/incidencerates/index.php, and http://www.cdc.gov/rdc/b1datatype/dt131.htm.

†† Additional information available at http://www.cdc.gov/cancer/npcr/uscs/technical_notes/interpreting/race.htm.
